# Health evaluation of African penguins (*Spheniscus demersus*) in southern Africa

**DOI:** 10.4102/ojvr.v83i1.1147

**Published:** 2016-09-20

**Authors:** Nola J. Parsons, Tertius A. Gous, Adam M. Schaefer, Ralph E.T. Vanstreels

**Affiliations:** 1 Southern African Foundation for the Conservation of Coastal Birds, Bloubergrant, South Africa; 2 Bayworld Centre for Research and Education, Port Elizabeth, South Africa; 3 Veterinary Pathologist, Helderberg, South Africa; 4 Harbor Branch Oceanographic Institution, Florida Atlantic University, United States; 5 Laboratory of Wildlife Comparative Pathology, University of São Paulo, Brazil

## Abstract

The African penguin (*Spheniscus demersus*) is an endangered seabird that breeds along the coast of Namibia and South Africa, and disease surveillance was identified as a priority for its conservation. Aiming for the establishment of baseline data on the presence of potential pathogens in this species, a comprehensive health assessment (blood smear examination, haematology, biochemistry and serology) was conducted on samples obtained from 578 African penguins at 11 breeding colonies and a rehabilitation centre. There were 68 penguins that were seropositive for at least one of seven pathogens tested: avian encephalomyelitis virus, avian infectious bronchitis virus, avian reovirus, infectious bursal disease virus, Newcastle disease virus, *Mycoplasma gallisepticum* and *Mycoplasma synoviae.* All samples were seronegative for avian influenza virus subtypes H5 and H7 and infectious laryngotracheitis virus. The apparent prevalence of *Babesia* sp. and *Borrelia* sp. in blood smears was consistent with previous studies. *Babesia*-infected individuals had a regenerative response of the erythrocytic lineage, an active inflammatory response and hepatic function impairment. These findings indicate that African penguins may be exposed to conservation-significant pathogens in the wild and encourage further studies aiming for the direct detection and/or isolation of these microorganisms.

## Introduction

**FIGURE 1:**
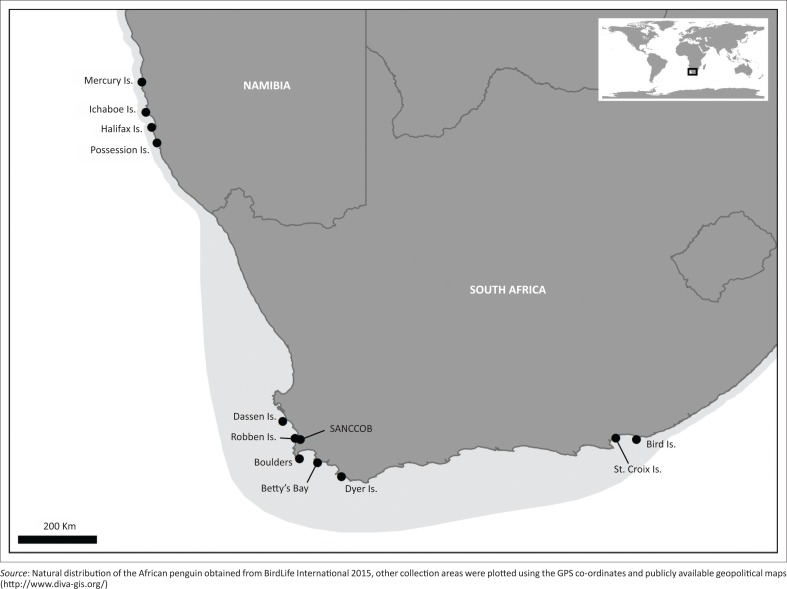
The natural distribution of the African penguin (light grey area) showing the sample collection sites (black dots).

The African penguin (*Spheniscus demersus*) is considered an endangered species (BirdLife International 2015) that breeds from central Namibia to South Africa’s Eastern Cape Province (Hockey, Dean & Ryan 2005) (Figure 1). There has been more than a 60% decrease in the population between 2001 and 2009, mainly attributable to changes in overall abundance and local availability of prey (Crawford *et al*. 2006, 2011; Sherley *et al*. 2013). The levels of breeding success were deemed inadequate to sustain the African penguin population, and among other conservation efforts, limiting mortality through controlling the spread of disease was suggested to try to maintain an equilibrium situation (Crawford *et al*. 2006).

Disease is a major ecological force that has the potential to cause significant effects especially in threatened populations (Friend, McLean & Dein 2001) and Heard *et al*. (2013) showed that the threat of disease increases with the level of extinction risk in all species. However, there is limited knowledge on the effects of disease on population dynamics of seabirds (Lewison *et al*. 2012) or even for the role of disease as a major threat to species at risk of extinction (Heard *et al*. 2013). While a single disease outbreak could decimate a population, the true cost of disease may be associated with chronic attrition of the population (Friend *et al*. 2001) and thereby influence metabolic rate, life history traits and social status (Barbosa & Palacios 2009).

Comprehensive health assessments of free-ranging avian species have rarely been reported in the literature (Smith *et al*. 2008). Modern conservation efforts can be enhanced by the availability of comprehensive health assessment data at a population level (Karesh & Cook 1995). Disease is often listed as a predicted threat to threatened species but this is generally a precautionary approach because there is a lack of surveillance data necessary to fully evaluate the threat (Heard *et al*. 2013). Therefore, health assessments and the compilation of baseline data on the presence of parasites and potential pathogens fill a critical data gap, particularly for endangered species. If a species is negatively affected by a major threat other than disease, that species is more likely to be simultaneously threatened by disease (Heard *et al*. 2013).

Several parasites have been recorded from the African penguin: seven trematode species, two nematode species, one argasidae tick species and two louse species (Brandão, Moreira & Luque 2014). Only the trematode *Cardiocephaloides physalis* has caused mortality in the African penguin (Randall & Bray 1983; Horne, Bray & Bousfield 2011); however, all parasite species may affect the fitness of the host, predispose the individual to disease, cause poor breeding productivity and nest desertion (Brandão *et al*. 2014; Duffy 1983; Kanarek, Horne & Zaleśny 2013).

A large-scale health assessment was conducted on the African penguin following the methods reported by Karesh *et al*. (1999), Smith *et al*. (2008) and Travis *et al*. (2006) on other penguin species, using blood smear examination, haematology, biochemistry and serology. Adult penguins in the breeding season on the colonies in South Africa as well as penguins admitted for rehabilitation were sampled. Additional samples included banked serum samples from penguins previously admitted to the Southern African Foundation for the Conservation of Coastal Birds (SANCCOB) and previous colony samples.

## Methods

### Sampling procedures

A total of 578 samples from the breeding range were analysed in this study. These samples were obtained from African penguins bled at Western Cape and Eastern Cape breeding colonies as well as from African penguins bled on admission for rehabilitation to SANCCOB (Cape Town, Western Cape); collected from various Namibian colonies in 2009 and from areas in the Western Cape from 2010 to 2013 (Figure 1). African penguins are admitted for rehabilitation because of oiling, debilitation, injuries, arrested moult and eggs and chicks admitted for hand-rearing (Parsons & Underhill 2005). Table 1 summarises the distribution of the sampling effort in relation to the sample collection site, month and year, clinical history and laboratory examinations.

**TABLE 1:** Summary of the sampling effort and tests evaluated during this health assessment of African penguins.

Group	Location	Sampling period	Clinical history	Sample size	Blood smear	Haematology	Serum chemistry	Serology	Sexing
Western Cape 2007–2008	Dassen Island	December 2007	Healthy	38	-	-	-	X	-
	Dassen Island	December 2008	Healthy	41	-	-	-	X	-
	Robben Island	December 2007	Healthy	17	-	-	-	X	-
	Robben Island	December 2008	Healthy	38	-	-	-	X	-
	Boulders	February 2008	Healthy	10	-	-	-	X	-
	Dyer Island	January 2008	Healthy	23	-	-	-	X	-
	Dyer Island	December 2008	Healthy	38	-	-	-	X	-
Namibia 2009	Halifax Island	April 2009	Oiled	9	X	-	-	X	X
	Ichaboe Island	April 2009	Oiled	12	X	-	-	X	X
	Mercury Island	April 2009	Oiled	15	X	-	-	X	X
	Possession Island	April 2009	Oiled	5	X	-	-	X	X
Western Cape 2010–2012	Dassen Island	December 2011	Healthy	20	X	X	X	X	X
	Robben Island	June 2011	Healthy	20	X	X	X	X	X
	Boulders	August 2012	Healthy	20	X	X	X	X	X
	Betty’s Bay	August 2010	Healthy	20	X	X	X	X	X
	Dyer Island	August 2010	Healthy	20	X	X	X	X	X
Eastern Cape 2012	Bird Island	July 2012	Healthy	50	X	X	X	X	X
	St. Croix Island	July 2012	Healthy	17	X	X	X	X	X
Rehabilitation 2010–2013	SANCCOB	2010–2013	Oiled	60	X	X	X	X	X
	SANCCOB	2010–2013	Chick	53	X	X	X	X	X
	SANCCOB	2010–2013	Moulting	17	X	X	X	X	X
	SANCCOB	2010–2013	Weak or wounded	35	X	X	X	X	X

SANCCOB, Southern African Foundation for the Conservation of Coastal Birds.

Colony samples were collected from penguins that were visually healthy on examination by a veterinarian. All penguins sampled at breeding colonies were adults, with the exception of 11 chicks and 1 juvenile sampled in the Western Cape 2007–2008 group. Birds selected were either resting in the colony or sitting on nests with medium to large chicks. Handling time was 5–10 min per bird, and the birds were released near their nest sites. Samples collected from penguins in the Namibia 2009 group were obtained on the second day of admission to the centre, and all were adults. Samples collected from penguins in the rehabilitation 2010–2013 group were obtained within the first 3 days of admission to the centre and comprised 53 chicks, 25 juveniles and 87 adults. For 365 of the total samples, sex was determined through routine DNA analysis by Molecular Diagnostic Services (Pty) Ltd (Durban, South Africa): 186 male penguins (51%) and 179 female penguins (49%).

### Haematology

Blood (5 mL – 20 mL) was collected through veni-puncture of the jugular vein using a 21-G needle (25 mm × 0.8 mm), immediately transferred into ethylenediaminetetraaceticacid and serum clot activator tubes (Vacuette^®^; Greiner Bio-One, Austria) and stored at 4 °C for up to 60 h until being analysed. Serum clot activator tubes were centrifuged and serum transferred into separate eppendorf tubes and immediately frozen at -20 °C. Blood smears were prepared, air-dried, fixed in methanol and stained with modified Wright–Giemsa stain (Kyro-Quick^®^; Kyron Laboratories [Pty] Ltd, Benrose, South Africa). All slides were examined for blood parasites for 10 min using a 50× oil immersion lens with a 10× eyepiece. Haematology and biochemistry analyses were performed following routine laboratory procedures at IDEXX Laboratories (Pty) Ltd (Cape Town, South Africa) (see Parsons *et al*. 2015b for details).

### Serology

The frozen serum samples were submitted to the Western Cape Provincial Veterinary Laboratory (Stellenbosch, South Africa) for haemagglutination inhibition assay (HIA) for avian influenza virus subtypes H5 and H7 (AIV H5, AIV H7) and Newcastle disease virus (NDV) and for serum plate agglutination (SPA) testing for *Mycoplasma gallisepticum* (MG) and *Mycoplasma synoviae* (MS). The HIA testing for avian influenza virus was done according to the protocol for non-chicken species (World Organisation for Animal Health 2014). Additionally, samples were submitted to IDEXX Laboratories (Pty) Ltd (Johannesburg, South Africa) for indirect enzyme-linked immunosorbent assay (ELISA) testing for avian infectious bronchitis virus (IBV), avian encephalomyelitis virus (AEV), avian reovirus (ARV), infectious bursal disease virus (IBDV), MG and MS (Table 2). ELISA testing used secondary antibodies targeting chicken IgY. In the case of *Mycoplasma* spp., SPA and ELISA were used to test different subsets of samples. Because of the occurrence of herpesvirus respiratory infections at the same facility (Parsons *et al*. 2015a), a limited number of samples were submitted to Agrilabs (Pioneerfoods [Pty] Ltd, Malmesbury, South Africa) to be tested for infectious laryngotracheitis virus (ILTV, also referred to as gallid herpesvirus 1) through indirect ELISA.

**TABLE 2:** Diagnostic results for pathogens tested during this health assessment of African penguins.

Pathogen	Test	Western Cape 2007–2008		Western Cape 2010–2012		Eastern Cape 2012		Namibia spill 2009		Rehabilitation 2010–2013		Total	
		%	*n*	%	*n*	%	*n*	%	*n*	%	*n*	%	*n*
**Serology**													
Avian encephalomyelitis virus	ELISA	0.93	107	5.00	100	0.00	65	10.00	20	3.31	151	2.93	443
Avian infectious bronchitis virus	ELISA	6.54	107	0.00	100	0.00	65	40.00	20	0.66	151	3.61	443
Avian Influenza Virus subtype H5	HIA	0.00	98	0.00	100	0.00	67	0.00	21	0.00	153	0.00	439
Avian Influenza Virus subtype H7	HIA	0.00	98	0.00	100	0.00	67	0.00	21	0.00	153	0.00	439
Avian reovirus	ELISA	2.80	107	0.00	100	0.00	65	5.00	20	0.00	151	0.90	443
Infectious bursal disease virus	ELISA	4.67	107	2.00	100	0.00	65	15.00	20	1.32	151	2.71	443
Infectious laryngotracheitis virus	ELISA	Not tested	Not tested	0.00	37	Not tested	Not tested	Not tested	Not tested	0.00	36	0.00	73
Newcastle Disease Virus	HIA	2.04	98	3.00	100	0.00	67	0.00	21	0.00	153	1.14	439
*Mycoplasma gallisepticum*	SPA	5.26	95	22.64	53	Not tested	Not tested	0.00	21	3.08	65	8.12	234
*Mycoplasma gallisepticum*	ELISA	Not tested	Not tested	2.50	40	0.00	65	Not tested	Not tested	0.00	84	0.53	189
*Mycoplasma synoviae*	SPA	9.47	95	1.72	58	Not tested	Not tested	0.00	21	0.00	65	4.18	239
*Mycoplasma synoviae*	ELISA	Not tested	Not tested	2.50	40	0.00	65	Not tested	Not tested	0.00	84	0.53	189
**Total**		**20.00**	**205**	**25.00**	**100**	**0.00**	**67**	**34.15**	**41**	**6.06**	**165**	**11.76**	**578**
**Blood smear examination**													
*Babesia* sp.	Blood smear	Not tested	Not tested	3.00	100	1.52	66	2.44	41	17.68	164	9.16	371
*Borrelia* sp.	Blood smear	Not tested	Not tested	0.00	100	0.00	66	0.00	41	1.83	164	0.81	371

SPA, serum plate agglutination; HIA, haemagglutination inhibition assay; ELISA, enzyme-linked immunosorbent assay.

### Data analysis

Statistical significance was set at 0.05 and tests were two-tailed using SPSS 21 for Windows (IBM Corp., 2011, Armonk, NY, USA). Fisher’s exact test was used to evaluate if the seroprevalence (number of positive samples/number of samples tested) for *Mycoplasma* spp. was different in relation to the serological test (SPA or ELISA). The data set presented by Parsons *et al*. (2015b) was used as haematological reference values for comparison with seropositive individuals; this data set comprises the seronegative and blood parasite-negative, apparently healthy adult African penguins sampled at colonies in this study. Mann-Whitney tests were used to compare haematological results between reference values and individuals that were seropositive for AEV, MG (SPA test) or two or more pathogens. Haematological results of individuals seropositive for other pathogens were not included in this analysis because of insufficient sample size (less than five samples).

Because *c.* 60% of the blood parasite-positive individuals were chicks, a different data set had to be used as reference values to evaluate the haematological results of these individuals; seronegative and blood parasite-negative apparently healthy African penguin chicks admitted to SANCCOB were used as a reference data set. Mann–Whitney tests were used to compare haematological results between these reference values and individuals positive for *Babesia* sp. On the other hand, *Borrelia* sp.–positive and mixed infection–positive individuals were not included in this analysis because of insufficient sample size.

## Results

A total of 578 individuals were screened; of those, 68 penguins were seropositive for at least one of the nine pathogens tested (Table 2); of these, 12 individuals were seropositive for more than one of the diseases tested: AEV + IBDV (2 samples), AEV + IBV (1), AEV + IBDV + IBV (1), AEV + MG (1), ARV + IBV (1), IBDV + IBV (2), IBDV + MS (1), MG + MS (2), and MG + NDV (1). All samples were seronegative for AIV H5, AIV H7 and ILTV. Samples tested for antibodies against *Mycoplasma* spp. using SPA were more frequently positive (4.2% for MG and 8.1% for MS) than those tested using ELISA (0.5% for both MG and MS); this occurred for both MG (*p* < 0.01) and MS (*p* = 0.03). Haematological results for seropositive individuals are presented in Table 3.

**TABLE 3:** Morphometry, haematology and serum chemistry results of seropositive individuals, in relation to reference values for healthy adult wild African penguins.

Parameter	Unit	Reference values (healthy adults)		Avian encephalomyelitis virus		*Mycoplasma gallisepticum* (serum plate agglutination test)		Two or more pathogens		Infectious bursal disease virus		Infectious bronchitis virus		Newcastle disease virus	
		mean ± s.d.	*n*	mean ± s.d.	*n*	mean ± s.d.	*n*	mean ± s.d.	*n*	mean ± s.d.	*n*	mean ± s.d.	*n*	mean ± s.d.	*n*
Head length	mm	121.1 ± 3.9	108	120.0 ± 5.9	7	120.1 ± 3.2	12	118.4 ± 7.4	6	127.2	1	117.9	1	118.3	2
Body mass	kg	2.86 ± 0.37	108	2.62 ± 0.40	8*	2.76 ± 0.30	12	2.71 ± 0.50	10	2.84	1	2.43 ± 0.10	5*	2.62	2
Haematocrit	%	46.0 ± 5.7	107	46.1 ± 4.6	8	45.1 ± 3.3	12	46.6 ± 7.9	10	38.0	1	50.6 ± 3.5	5	51.0	2
Haemoglobin	g/dL	18.4 ± 2.4	103	19.2 ± 0.6	6	18.9 ± 1.8	12	19.1 ± 1.6	5	16.1	1	19.3	1	19.3	2
Red blood cell count	10^12^/L	1.82 ± 0.26	103	1.94 ± 0.09	6	1.83 ± 0.19	12	1.93 ± 0.15	5	1.65	1	1.99	1	1.88	2
MCV	fL	251.0 ± 35.6	103	243.1 ± 13.9	6	248.5 ± 27.8	12	246.3 ± 13.1	5	230.3	1	236.2	1	271.9	2
MCH	pg	101.1 ± 14.3	103	99.0 ± 2.7	6	104.0 ± 11.5	12	98.8 ± 4.6	5	97.6	1	97.0	1	102.7	2
MCHC	g/dL	40 ± 3.5	103	40.8 ± 1.5	6	41.9 ± 1.9	12	40.2 ± 2.1	5	42.4	1	41.1	1	37.8	2
White blood cell count	10^9^/L	17.7 ± 8.4	103	13.0 ± 8.1	6	20.2 ± 4.5	12	17.0 ± 3.7	5	30.0	1	14.2	1	22.4	2
Sodium	mmol/L	154 ± 6	105	146 ± 9	7*	145 ± 5	12*	151 ± 5	6	148	1	146	1	158	1
Potassium	mmol/L	5.09 ± 2.52	105	5.00 ± 1.48	7	10.05 ± 4.39	12*	4.85 ± 1.12	6	6.20	1	5.86	1	3.65	1
Chloride	mmol/L	121 ± 6	104	115 ± 7	7*	115 ± 5	12*	116 ± 4	6*	114	1	109	1	128	1
Calcium	mmol/L	2.77 ± 0.82	105	2.53 ± 0.19	7	2.43 ± 0.24	12	2.50 ± 0.09	6	1.96	1	2.35	1	2.58	1
Inorganic phosphate	mmol/L	1.53 ± 0.62	105	1.35 ± 0.46	7	1.65 ± 1.03	12	1.48 ± 0.55	6	1.20	1	1.80	1	1.69	1
Creatinine	mmol/L	24.1 ± 11.9	105	20.0 ± 17.0	7	12.6 ± 9.9	12*	16.8 ± 8.5	6*	5.0	1	4.0	1	16.0	1
Cholesterol		mmol/L	5.36 ± 1.36	105	5.41 ± 1.20	7	6.12 ± 1.37	12	5.32 ± 0.67	6	5.70	1	4.30	1	6.10	1
Glucose	mmol/L	11.8 ± 2.2	105	12.5 ± 1.4	7	12.6 ± 1.2	12	12.2 ± 1.5	6	11.1	1	11.9	1	10.0	1
Uric Acid	mmol/L	394 ± 221	104	604 ± 482	7	345 ± 280	11	539 ± 186	6	73	1	448	1	263	1
Bile Acids	mmol/L	9.53 ± 16.75	87	18.26 ± 15.33	5	9.26 ± 13.30	9	17.54 ± 21.15	5	1.64	1	7.20	1	23.56	2
Total serum protein	g/L	59.0 ± 9.6	105	53.7 ± 7.0	7	70.0 ± 5.8	12	61.8 ± 8.6	6	53.7	1	51.0	1	49.0	1
Albumin	g/L	19.3 ± 4.0	105	16.6 ± 3.2	7	21.0 ± 3.2	12	20.5 ± 3.0	6	16.6	1	16.0	1	15.0	1
Globulin	g/L	39.8 ± 6.3	105	37.1 ± 3.9	7	49.0 ± 3.5	12	41.3 ± 5.8	6	37.1	1	35.0	1	35.0	1
Albumin / globulin	-	0.48 ± 0.05	105	0.44 ± 0.05	7	0.50 ± 0.06	12	0.46 ± 0.04	6	0.46	1	0.43	1	0.43	1
Aspartate transaminase	U/L	218 ± 90	104	293 ± 235	7	247 ± 107	12	184 ± 44	6	151	1	326	1	120	1
Creatine kinase	U/L	419 ± 272	105	461 ± 498	7	561 ± 358	12	365 ± 187	6	188	1	375	1	385	1

*Source:* Reference values obtained from Parsons *et al*. (2015b); all other values from this study

MCV, mean corpuscular volume; MCH, mean corpuscular haemoglobin; MCHC, mean corpuscular haemoglobin concentration.

* indicate groups that were significantly different from the reference values (only evaluated when sample size was ≥ 5).

Blood smears revealed 33 samples were positive for *Babesia* sp., 2 individuals were positive for *Borrelia* sp. and 1 individual was positive for both *Babesia* sp. and *Borrelia* sp. (Table 2); no other blood parasites were observed. These blood parasites were morphologically consistent with those documented by Earlé *et al*. (1993) and Yabsley *et al*. (2012). Only two blood smear–positive individuals (*Babesia*-positive) were also found to be seropositive: one was seropositive to MG (SPA test) and the other was seropositive to both AEV and IBDV. Haematological results for blood smear–positive individuals are presented in Table 4.

**TABLE 4:** Morphometry, haematology and serum chemistry results of blood smear–positive individuals, in relation to reference values for healthy African penguin chicks admitted to the Southern African Foundation for the Conservation of Coastal Birds (this study) and healthy adult wild African penguins.

Parameter	Unit	Reference values				*Babesia* sp.				*Borrelia* sp.		Co-infection by *Babesia* sp. and *Borrelia* sp.	
		Healthy chicks		Healthy adults		Chicks		Juveniles and adults					
		mean ± s.d.	*n*	mean ± s.d.	*n*	mean ± s.d.	*n*	mean ± s.d.	*n*	mean ± s.d.	*n*	mean ± s.d.	*n*
Head length	mm	106.7 ± 4.8	30	121.1 ± 3.9	108	103.9 ± 5.2	20*	119.3 ± 4.3	12	104.1	2	99.3	1
Body mass	kg	2.32 ± 0.30	30	2.86 ± 0.37	108	2.20 ± 0.20	20	2.36 ± 0.50	13*	1.67	2	2.02	1
Haematocrit	%	31.7 ± 6.4	30	46.0 ± 5.7	107	29.3 ± 5.0	20	40.5 ± 10.7	13	35.5	2	25.0	1
Haemoglobin	g/dL	12.6 ± 1.9	22	18.4 ± 2.4	103	9.3 ± 2.3	13*	15.1 ± 5.4	11*	12.2	1	8.2	1
Red blood cell count	10^12^/L	1.53 ± 0.18	22	1.82 ± 0.26	103	1.15 ± 0.32	13*	1.65 ± 0.37	11	1.61	1	1.00	1
MCV	fL	220.8 ± 14.7	22	251.0 ± 35.6	103	275.5 ± 41.6	13*	235.0 ± 30.3	11	211.2	1	250.0	1
MCH	pg	81.8 ± 4.8	22	101.1 ± 14.3	103	81.5 ± 4.3	13	88.3 ± 19.3	11*	75.8	1	82.0	1
MCHC	g/dL	37.2 ± 3.0	22	40.0 ± 3.5	103	30.1 ± 4.1	13*	37.2 ± 5.3	11*	35.9	1	32.8	1
White blood cell count	10^9^/L	15.0 ± 5.2	22	17.7 ± 8.4	103	21.2 ± 5.0	13*	31.4 ± 23.4	11*	24.6	1	14.2	1
Sodium	mmol/L	146 ± 5	27	154 ± 6	105	148 ± 3	19*	144 ± 13	11*	140	2	139	1
Potassium	mmol/L	5.45 ± 0.71	27	5.09 ± 2.52	105	5.67 ± 0.74	19	5.57 ± 1.59	9*	4.91	2	5.03	1
Chloride	mmol/L	113 ± 5	27	121 ± 6	104	115 ± 3	19	112 ± 9	11*	108	1	105	1
Calcium	mmol/L	2.58 ± 0.15	27	2.77 ± 0.82	105	2.57 ± 0.11	19	2.38 ± 0.35	11	2.53	2	2.34	1
Inorganic phosphate	mmol/L	1.89 ± 0.28	26	1.53 ± 0.62	105	2.31 ± 0.49	19*	2.08 ± 1.55	11	2.35	2	1.73	1
Creatinine	mmol/L	17.6 ± 12.6	27	24.1 ± 11.9	105	20.6 ± 8.4	19	41.6 ± 44.8	11	41.5	2	22.0	1
Cholesterol	mmol/L	4.45 ± 1.03	27	5.36 ± 1.36	105	4.61 ± 0.96	19	4.95 ± 2.13	11	5.00	2	2.70	1
Glucose	mmol/L	12.3 ± 1.3	27	11.8 ± 2.2	105	12.2 ± 1.0	19	10.4 ± 4.7	11	10.9	2	13.0	1
Uric Acid	mmol/L	652 ± 319	27	394 ± 221	104	542 ± 436	18*	562 ± 488	11	460	2	608	1
Bile Acids	mmol/L	25.47 ± 14.25	22	9.53 ± 16.75	87	14.19 ± 11.20	19	9.16 ± 13.37	11	43.10	2	12.39	1
Total serum protein	g/L	43.1 ± 5.4	25	59.0 ± 9.6	105	46.3 ± 4.6	19*	48.2 ± 17.7	11	45.5	2	34.0	1
Albumin	g/L	13.7 ± 1.4	27	19.3 ± 4.0	105	14.1 ± 1.6	19	15.5 ± 5.8	11*	13.5	2	10.0	1
Globulin	g/L	29.6 ± 4.4	25	39.8 ± 6.3	105	31.8 ± 4.0	19	32.7 ± 12.3	11	32.0	2	24.0	1
Albumin / globulin	-	0.47 ± 0.05	25	0.48 ± 0.05	105	0.45 ± 0.04	19	0.49 ± 0.10	11	0.42	2	0.42	1
Aspartate transaminase	U/L	145 ± 38	27	218 ± 90	104	151 ± 38	19	376 ± 247	11	797	2	120	1
Creatine kinase	U/L	392 ± 124	27	419 ± 272	105	368 ± 153	19	1275 ± 2283	11*	1559	2	250	1

*Source:* Reference values for adults obtained from Parsons *et al*. (2015b), all other values from this study

MCV, mean corpuscular volume; MCH, mean corpuscular haemoglobin; MCHC, mean corpuscular haemoglobin concentration.

* indicate groups that were significantly different from the reference values of their age class (only evaluated when the sample size was ≥ 5).

Of the positive individuals, 66 (97%) were adults compared to two (3%) chicks. There was no difference across genders. Of the positive adults, there were 49 (74%) that were sampled as healthy individuals in wild colonies (90% unknown breeding status, 10% sitting with chicks) and 17 (26%) sampled when admitted for rehabilitation (94% oiled, 6% injured). There was a significant difference in the prevalence of seropositive individuals between the three geographical areas: Namibia, Western Cape and Eastern Cape. Complete details on the sampling effort and serological and blood smear results in relation to age group and sex are provided in Table 1-A1, and in relation to breeding colony in Table 2-A1.

## Ethical considerations

Research permits to conduct this work were obtained by the Department of Environmental Affairs (DEA) (RES2012/61 EXT, RES2011/19, and RES2010/58), CapeNature (AAA007-00047-0056, AAA004-0508-0035, AAA004-000120-0035 and AAA007-00040-0035) and South African National Parks (PARSN1027). Procedures were approved by the Animal Ethics Committee of the DEA, and all blood samples were collected by veterinarians (N.J.P., T.A.G.) registered with the South African Veterinary Council. Where applicable, ARRIVE guidelines for reporting *in vivo* animal experiments (Kilkenny *et al*. 2010) have been adhered to.

## Discussion

Our results should be interpreted taking into account the characteristics and inherent limitations of the serological tests used in this study. Because serological tests specifically designed for African penguins are not currently available, we used commercial tests designed for poultry. The indirect ELISA tests used in this study rely on the basic assumption that antibodies against chicken IgY can also reliably recognise penguin IgY. While these specific commercial tests have not undergone thorough validation to estimate their sensitivity and specificity when applied to samples from African penguins, other studies on the antigenic properties of penguin immunoglobulins corroborate the validity of their basic methodological assumption (Bizelli *et al*. 2015; Graczyk *et al*. 1994, 1995). Unfortunately, the lack of serological tests specifically designed or validated for penguins is a recurrent methodological limitation of serological inquiries in these species (Karesh *et al*. 1999; Nunes *et al*. 2012; Smith *et al*. 2008; Travis *et al*. 2006; Uhart *et al*. 2004), which hopefully will be overcome through ongoing research aiming at the production of secondary antibodies specifically targeting penguin IgY (Bizelli *et al*. 2015). On the other hand, the HIA used to test for NDV, AIV H5 and AIV H7 is not subject to this limitation because it does not rely on the recognition by secondary antibodies.

It is also worth noting that this is not a comprehensive study into all pathogens and parasites that can affect the health of African penguins on an individual or population level. Further studies looking at epidemiology as well as interaction between parasites, pathogens and fitness of individuals are encouraged.

### Avian encephalomyelitis virus (Picornaviridae)

Seropositivity to AEV was identified in the Namibian and the Western Cape samples and in penguins admitted for rehabilitation at SANCCOB; overall seroprevalence was relatively low (2.9%). AEV has been documented in domestic birds in South Africa (Odend’hal 1983), but it has never been demonstrated to infect penguins by direct diagnostic methods. Serological surveys examining penguins in Peru and at the Falkland and Galapagos Islands have only found negative results (Smith *et al*. 2008; Travis *et al*. 2006; Uhart *et al*. 2004), whereas Karesh *et al*. (1999) found antibodies against AEV in southern rockhopper penguins (*Eudyptes chrysocome*) in Argentina, with seroprevalence (3%) similar to that observed in this study.

AEV infections seldom cause clinical disease in adult chickens, but can lead to significant decreases in egg production and hatchability; however, in young chickens, AEV can produce paralysis, ataxia and muscular dystrophy (Tannock & Shafren 1994). In this study, AEV seropositive penguins had slightly lower serum sodium and chloride concentrations; this cannot be explained by the pathogenesis of AEV infection and is therefore interpreted as an incidental finding.

### Avian infectious bronchitis virus (Coronaviridae)

Seropositivity to IBV was identified in the Namibian and the Western Cape samples and in penguins admitted for rehabilitation at SANCCOB; overall seroprevalence was relatively low (3.6%). Few studies have tested penguins for antibodies against IBV. Karesh *et al*. (1999) found a seroprevalence between 23% and 47% (depending on the titre cutpoint) in southern rockhopper penguins in Argentina, whereas Smith *et al*. (2008) did not detect antibodies against this pathogen in Humboldt penguins (*Spheniscus humboldti*) in Peru. DNA from coronaviruses has been detected in the tissues of beachcast carcasses of Magellanic penguins (*Spheniscus magellanicus*) in Brazil; however, it is unclear whether these viruses were associated with disease (Niemeyer *et al*. 2012).

Coronaviruses such as IBV are known to cause respiratory, intestinal and reproductive diseases in both domestic and wild birds (Gerlach 1994). However, the significance of this infection in penguins is unclear. Individuals that were seropositive for IBV had significantly lower body mass but not head length than otherwise healthy adults, suggesting poorer body condition compared to those that were seronegative. However, this result should be interpreted with caution considering the low sample size.

### Avian influenza virus (Orthomyxoviridae)

We found no serological evidence of highly pathogenic influenza A virus (subtypes H5 and H7), despite past evidence of their circulation in wild birds in South Africa (Abolnik *et al*. 2012; Cumming *et al*. 2011). Penguin seropositivity to AIV has been demonstrated by studies in the Antarctic (Abad *et al*. 2013; Morgan & Westbury 1981; Wallensten *et al*. 2006) and Subantarctic (Abad *et al*. 2013), and Hurt *et al*. (2014) have demonstrated that the AIV H11N2 present in penguins on the Antarctic Peninsula is an evolutionarily distinct lineage, not closely related to AIV strains from migratory flying birds. On the other hand, the few serological studies on penguins at lower latitudes conducted to date have failed to demonstrate exposure to AIV (Karesh *et al*. 1999; Smith *et al*. 2008; Travis *et al*. 2006). However, this is unlikely to result from an absence of circulation of these viruses, as their worldwide distribution has been extensively documented (Olsen *et al*. 2006). It is likely that these negative results reflect the fact that AIV occurrence is highly variable and species and location dependent (Hanson *et al*. 2008). It must also be considered that antibodies against AIV subtypes other than H5 and H7 would have gone undetected by the tests used in this study.

### Avian reovirus (Reoviridae)

Antibodies against ARV were detected in wild African penguins sampled in Namibia and the Western Cape, with a low overall seroprevalence (0.9%). Reovirus-like agents with some similarity to reference chicken reovirus strain were isolated in African penguins that died at a zoo in the United Kingdom (Gough *et al*. 2002). However, in that case, the birds were seronegative to the one-way neutralisation test, and it was unclear what role the virus played in their deaths (Gough *et al*. 2002). Surveys in Peru and on the Falkland and Galapagos Islands have found only seronegative penguins (Smith *et al*. 2008; Travis *et al*. 2006; Uhart *et al*. 2004). On the other hand, Karesh *et al*. (1999) detected antibodies against ARV in 23% of southern rockhopper penguins sampled in Argentina. ARV has been documented in domestic birds worldwide, including South Africa, and may lead to a broad variety of clinical presentations (Gerlach 1994; Van Loon *et al*. 2001).

### Infectious bursal disease virus (Birnaviridae)

Antibodies against IBDV were detected in wild African penguins sampled in Namibia and the Western Cape and in penguins admitted for rehabilitation at SANCCOB; overall seroprevalence was relatively low (2.7%). Antibodies against IBDV have been demonstrated in penguins by studies using ELISA in Brazil (Nunes *et al*. 2012) and virus neutralisation tests in Crozet Archipelago and at various locations in Antarctica (Gardner, Kerry & Riddle 1997; Gauthier-Clerc *et al*. 2002; Watts, Miller & Shellam 2009), whereas studies using agar-gel diffusion tests have failed to obtain positive results in South America (Karesh *et al*. 1999; Smith *et al*. 2008; Travis *et al*. 2006). Watts *et al*. (2009) argue that IBDV serotype 1 is endemic and widespread in Antarctic birds, with Emperor penguins (*Aptenodytes forsteri*) playing a key role in the virus’ persistence in Antarctica.

IBDV is known to cause disease in young chickens, in which it can produce bursal lymphoid depletion and high mortality (World Organisation for Animal Health 2008). No clinical signs of disease have been observed in any of the seropositive penguin species in the wild (Gardner *et al*. 1997; Gauthier-Clerc *et al*. 2002; Nunes *et al*. 2012; Watts *et al*. 2009). Gough *et al*. (2002) reported the isolation of IBDV serotype 2 from the tissues of African and Macaroni penguins (*Eudyptes chrysolophus*) deceased at a zoo in the United Kingdom and considered that although the infection was not primarily responsible for the deaths, it may have exacerbated concurrent disease conditions. Unfortunately, in this study, we did not have a sufficient number of seropositive penguins to evaluate the potential health effects of exposure to IBDV.

### Infectious laryngotracheitis virus (Herpesviridae)

There were no positive samples in serology testing for ILTV (also known as gallid herpesvirus 1) despite previous evidence that African penguins are susceptible to herpesvirus-like infections (Kincaid, Bunton & Cranfield 1988; Parsons *et al*. 2015a). Previous studies on other penguin species have also failed to identify antibodies against this virus (Karesh *et al*. 1999; Smith *et al*. 2008). Wild African penguin chicks have presented herpesvirus-like respiratory infections, which were not detected by molecular or serological tests targeting ILTV, suggesting that a different herpesvirus was involved (Parsons *et al*. 2015a).

### Newcastle disease virus (Paramyxoviridae)

Five individuals were seropositive to NDV (also known as avian paramyxovirus type 1), all of which were sampled in the Western Cape. Penguins that were seropositive for NDV have been demonstrated in the Antarctic (Morgan & Westbury 1981), Argentina (Karesh *et al*. 1999), Macquarie Island (Morgan *et al*. 1981) and South Shetland Islands (Thomazelli *et al*. 2010). Thomazelli *et al*. (2010) determined that the strains detected in penguins at the South Shetlands Islands had low pathogenicity. NDV infection has also been demonstrated in captive penguins in the United States (Pierson & Pfow 1975), where a velogenic neurotropic strain was identified, and in Israel (Haddas *et al*. 2014), where the pathogenicity of the strain could not be determined. It is clear that penguins are susceptible to this virus and that some NDV strains, presumably those with low pathogenicity, circulate in wild penguin populations. NDV has also been demonstrated in great white pelicans (*Pelecanus onocrotalus*) in the Western Cape (Assunção *et al*. 2007).

It is interesting to note that one of the individuals identified as seropositive was a penguin that had been rehabilitated at SANCCOB 7 years earlier and, at that time, received vaccination for NDV. The vaccination consisted of an initial ocular spray vaccination on admission to the centre with live Lasota strain (Nobilis^®^ ND LASOTA, Kempton Park, South Africa) followed by an intramuscular injection of inactivated Lasota strain (Lomovac, TAD, Germany) (N.J. Parsons, unpublished data). There is no literature, to our knowledge that determines how long the vaccination antibodies remain detectable in a penguin following vaccination. Although it is unlikely that antibodies are still circulating 7 years after vaccination, it is possible that vaccination may have interfered with the results. SANCCOB stopped routinely marking all penguins before release into the wild in August 2005, but routinely vaccinated for NDV up until August 2008.

### Mycoplasma spp.

Serological tests for MG and MS have not been routinely used in wild penguin species. There was inconsistency between the serological tests, with a higher frequency of positives when samples were tested with SPA compared to ELISA testing. While different subsets of samples were tested with each test, this discrepancy suggests an inherent difference in the sensitivity and specificity of the two tests. It is also important to consider that cross-reactivity with other *Mycoplasma* spp. from African penguins in this study is possible. Multiple *Mycoplasma* spp. (excluding MG and MS) have been demonstrated to occur in penguins (Banks, Cary & Hogg 2009; Buckle *et al*. 2013; Dewar *et al*. 2013; Frasca *et al*. 2005). Furthermore, Frasca *et al*. (2005) found cross-reactivity of antibodies against *Mycoplasma sphenisci* to antibodies against MG and MS in agglutination tests. Therefore, caution should be used when interpreting these results.

MG and MS potentially cause respiratory disease, sinusitis, conjunctivitis and synovitis in domestic and wild birds (Jordan 1975). *Mycoplasma sphenisci* was described in an African penguin showing signs of upper respiratory tract disease in a North American aquarium (Frasca *et al*. 2005) and *M. lipofaciens* was identified from the lungs of a Fiordland penguin (*Eudyptes pachyrhynchus*) after post-mortem examination showed bronchopneumonia (Buckle *et al*. 2013). On the other hand, *M. sphenisci* and other *Mycoplasma* spp. have been detected in the faeces of apparently healthy penguins in Antarctica and subantarctic islands (Banks *et al*. 2009; Dewar *et al*. 2013). In this study, African penguins seropositive to MG in the SPA test had considerably lower serum concentrations of sodium, chloride and creatinine and higher concentrations of potassium, suggesting impairment of kidney function. Although MG and MS are known to produce renal lesions, these tend to be less prominent than respiratory and articular lesions (Jordan 1975; Lockaby *et al*. 1998). Future studies will be necessary to identify which species of *Mycoplasma* occurs in African penguins and to confirm if it produces significant renal disease.

It is worth noting that great white pelicans have been shown to have high prevalence (98%) of *Mycoplasma* spp. in South Africa (Assunção *et al*. 2007). This species breeds sympatrically with and often predates on African penguins (Mwema, de Ponte Machado & Ryan 2010). Furthermore, because great white pelicans are known to feed on avian offal in agricultural areas (Crawford, Cooper & Dyer 1995), they could play a key role in spreading pathogens such as *Mycoplasma* spp. from domestic animals to seabirds (Assunção *et al*. 2007).

### Babesia sp. and Borrelia sp.

Although we did not fully characterise the blood parasites, their morphology was consistent with *Babesia peircei* and relapsing fever *Borrelia* as previously described in African penguins in the same region (Earlé *et al*. 1993; Yabsley *et al*. 2012). The apparent prevalence of *Babesia* sp. in wild African penguins in this study (1.5% – 3.0%) is similar to that observed in previous studies, as is the higher frequency of *Babesia* sp. and *Borrelia* sp. among chicks and individuals undergoing rehabilitation (Brossy *et al*. 1999; Earlé *et al*. 1993; Yabsley *et al*. 2012).

The pathological significance of *Babesia* sp. to penguins is not clear, and so far, this parasite has only been associated with only mild regenerative anaemia (Brossy *et al*. 1999; Cunningham *et al*. 1993; Vanstreels *et al*. 2015). In this study, African penguin chicks with *Babesia* sp. had significantly different haematological and serum chemistry values compared to healthy chicks. *Babesia*-infected penguins had abnormalities in erythrocyte size and lower haemoglobin concentration, suggesting a regenerative response of the erythrocytic lineage, presumably to the haemolysis caused by the parasite. Higher white blood cell counts in *Babesia*-infected penguins indicate an active inflammatory response to the parasite and/or a stress response. Finally, higher serum levels of creatinine kinase and lower serum levels of uric acids and albumin indicate impairment of hepatic function and may also be partly related to haemolysis (see Harrison & Lightfoot 2006).

## Conclusion

Considering the decreasing trend of the African penguin population (Crawford *et al*. 2011), disease is yet another significant threat to the species in addition to poor nutrition, environmental degradation and anthropogenic impacts (Woods *et al*. 2009). Serological surveillance can be a powerful tool to track the prevalence of pathogens that are otherwise difficult to detect in wildlife populations (Gilbert *et al*. 2013). The reported seroprevalence in this study is consistent with previously reported studies on wild penguins, suggesting that these are endemic pathogens or natural, apathogenic flora. It must also be borne in mind that the presence of antibodies indicates past exposure to a pathogen and does not necessarily indicate presence of the organism or active infection. In addition, cross-reaction of tests with other antigens and microorganisms may interfere with specificity of the results (Barbosa & Palacios 2009). Studies addressing the direct detection and isolation of pathogenic organisms in penguins are encouraged and, in combination with serological investigations, should provide deeper insight on their epidemiology in these birds.

## Appendix 1

**TABLE 1-A1:** Details of sampling effort and serological results in relation to age group and sex.

Group	Age	Sex	Infectious bronchitis virus		Avian encephalomyelitis virus		Avian reovirus		AIV H5	AIV H7	Infectious bursal disease virus		Infectious laryngotracheitis virus	Newcastle disease virus		*Mycoplasma gallisepticum* (SPA)		*Mycoplasma gallisepticum* (ELISA)		*Mycoplasma synoviae* (SPA)		*Mycoplasma synoviae* (ELISA)		*Babesia* sp.		*Borrelia* sp.	
			*n*	Positive *n*	*n*	Positive *n*	*n*	Positive *n*			*n*	Positive *n*		*n*	Positive *n*	*n*	Positive *n*	*n*	Positive *n*	*n*	Positive *n*	*n*	Positive *n*	*n*	Positive *n*	*n*	Positive *n*
Western Cape 2007–2008	Chick	Unknown	4	-	4	-	4	-	7	7	4	-	0	7	-	7	-	0	-	7	1	0	-	0	-	0	-
	Juvenile	Unknown	1	-	1	-	1	-	0	0	1	-	0	0	-	0	-	0	-	0	-	0	-	0	-	0	-
	Adult	Unknown	102	7	102	1	102	3	91	91	102	5	0	91	2	88	5	0	-	88	8	0	-	0	-	0	-
Namibia 2009	Adult	Male	10	5	10	1	10	1	9	9	10	2	0	9	-	9	-	0	-	9	-	0	-	19	-	19	-
		Female	10	3	10	1	10	-	11	11	10	1	0	11	-	11	-	0	-	11	-	0	-	21	1	21	-
		Unknown	0	-	0	-	0	-	1	1	0	-	0	1	-	1	-	0	-	1	-	0	-	1	-	1	-
Western Cape 2010–2012	Adult	Male	53	-	53	5	53	-	53	53	53	2	23	53	1	28	5	20	1	32	-	20	1	53	1	53	-
		Female	47	-	47	-	47	-	47	47	47	-	14	47	2	25	7	20	-	26	1	20	-	47	2	47	-
Eastern Cape 2012	Adult	Male	26	-	26	-	26	-	26	26	26	-	0	26	-	0	-	26	-	0	-	26	-	26	-	26	-
		Female	37	-	37	-	37	-	39	39	37	-	0	39	-	0	-	37	-	0	-	37	-	39	1	39	-
		Unknown	2	-	2	-	2	-	2	2	2	-	0	2	-	0	-	2	-	0	-	2	-	1	-	1	-
Rehabilitation 2010–2013	Chick	Male	24	-	24	-	24	-	25	25	24	-	19	25	-	14	-	10	-	15	-	10	-	31	13	31	-
		Female	18	-	18	-	18	-	16	16	18	-	17	16	-	11	-	4	-	12	-	4	-	20	7	20	2
		Unknown	1	-	1	1	1	-	1	1	1	1	0	1	-	0	-	1	-	0	-	1	-	1	1	1	-
	Juvenile	Male	14	-	14	-	14	-	14	14	14	-	0	14	-	8	-	6	-	8	-	6	-	14	3	14	-
		Female	10	-	10	-	10	-	11	11	10	-	0	11	-	4	-	7	-	4	-	7	-	11	1	11	1
	Adult	Male	41	-	41	1	41	-	42	42	41	1	0	42	-	10	-	31	-	9	-	31	-	43	1	43	-
		Female	39	1	39	3	39	-	40	40	39	-	0	40	-	18	-	21	-	17	-	21	-	40	3	40	-
		Unknown	4	-	4	-	4	-	4	4	4	-	0	4	-	0	-	4	-	0	-	4	-	4	-	4	-

SPA, serum plate agglutination; ELISA, enzyme-linked immunosorbent assay.

**TABLE 2-A1:** Details of sampling effort and serological results in relation to sampling location and/or clinical history.

Group	Location or clinical history	Infectious bronchitis virus		Avian encephalomyelitis virus		Avian reovirus		AIV H5	AIV H7	Infectious bursal disease virus		Infectious laryngotracheitis virus	Newcastle disease virus		*Mycoplasma gallisepticum* (SPA)		*Mycoplasma gallisepticum* (ELISA)		*Mycoplasma synoviae* (SPA)		*Mycoplasma synoviae* (ELISA)		*Babesia* sp.		*Borrelia* sp.	
		*n*	Positive *n*	*n*	Positive *n*	*n*	Positive *n*			*n*	Positive *n*		*n*	Positive *n*	*n*	Positive *n*	*n*	Positive *n*	*n*	Positive *n*	*n*	Positive *n*	*n*	Positive *n*	*n*	Positive *n*
Western Cape 2007–2008	Dassen Island	41	2	41	-	41	2	38	38	41	1	0	38	1	37	2	0	-	37	6	0	-	0	-	0	-
	Robben Island	27	5	27	1	27	1	28	28	27	1	0	28	-	28	-	0	-	28	2	0	-	0	-	0	-
	Boulders	0	-	0	-	0	-	10	10	0	-	0	10	1	9	-	0	-	9	-	0	-	0	-	0	-
Namibia 2009	Dyer Island	39	-	39	-	39	-	22	22	39	3	0	22	-	21	3	0	-	21	1	0	-	0	-	0	-
	Halifax Island	3	-	3	-	3	-	6	6	3	-	0	6	-	6	-	0	-	6	-	0	-	9	-	9	-
	Ichaboe Island	8	2	8	1	8	1	4	4	8	-	0	4	-	4	-	0	-	4	-	0	-	12	1	12	-
	Mercury Island	7	6	7	1	7	-	8	8	7	3	0	8	-	8	-	0	-	8	-	0	-	15	-	15	-
	Possession Island	2	-	2	-	2	-	3	3	2	-	0	3	-	3	-	0	-	3	-	0	-	5	-	5	-
Western Cape 2010–2012	Dassen Island	20	-	20	1	20	-	20	20	20	-	0	20	-	0	-	20	-	0	-	20	-	20	1	20	-
	Robben Island	20	-	20	-	20	-	20	20	20	-	0	20	-	18	8	0	-	20	-	0	-	20	1	20	-
	Boulders	20	-	20	4	20	-	20	20	20	2	0	20	-	0	-	20	1	0	-	20	1	20	-	20	-
	Betty’s Bay	20	-	20	-	20	-	20	20	20	-	18	20	2	19	1	0	-	20	-	0	-	20	-	20	-
	Dyer Island	20	-	20	-	20	-	20	20	20	-	19	20	1	16	3	0	-	18	1	0	-	20	1	20	-
Eastern Cape 2012	Bird Island	48	-	48	-	48	-	50	50	48	-	0	50	-	0	-	48	-	0	-	48	-	50	1	50	-
	St. Croix Island	17	-	17	-	17	-	17	17	17	-	0	17	-	0	-	17	-	0	-	17	-	16	21	16	-
Rehabilitation 2010–2013	Oiled	59	1	59	3	59	-	59	59	59	1	0	59	-	29	2	26	-	29	-	29	-	60	1	60	-
	Chick	43	-	43	1	43	-	42	42	43	1	36	42	-	15	-	27	-	15	-	15	-	52	-	52	2
	Moulting	16	-	16	-	16	-	17	17	16	-	0	17	-	14	-	3	-	14	-	14	-	17	3	17	-
	Weak/wounded	33	-	33	1	33	-	35	35	33	-	0	35	-	26	-	9	-	26	-	26	-	35	4	35	1

SPA, serum plate agglutination; ELISA, enzyme-linked immunosorbent assay.
